# Alzheimer’s Disease Associated Genes Ankyrin and Tau Cause Shortened Lifespan and Memory Loss in *Drosophila*

**DOI:** 10.3389/fncel.2019.00260

**Published:** 2019-06-11

**Authors:** James P. Higham, Bilal R. Malik, Edgar Buhl, Jennifer M. Dawson, Anna S. Ogier, Katie Lunnon, James J. L. Hodge

**Affiliations:** ^1^School of Physiology, Pharmacology and Neuroscience, University of Bristol, Bristol, United Kingdom; ^2^University of Exeter Medical School, University of Exeter, Exeter, United Kingdom

**Keywords:** Alzheimer’s disease, *Drosophila*, memory, lifespan, locomotion, neurodegeneration, Tau, Ankyrin

## Abstract

Alzheimer’s disease (AD) is the most common form of dementia and is characterized by intracellular neurofibrillary tangles of hyperphosphorylated Tau, including the 0N4R isoform and accumulation of extracellular amyloid beta (Aβ) plaques. However, less than 5% of AD cases are familial, with many additional risk factors contributing to AD including aging, lifestyle, the environment and epigenetics. Recent epigenome-wide association studies (EWAS) of AD have identified a number of loci that are differentially methylated in the AD cortex. Indeed, hypermethylation and reduced expression of the *Ankyrin 1* (*ANK1*) gene in AD has been reported in the cortex in numerous different post-mortem brain cohorts. Little is known about the normal function of ANK1 in the healthy brain, nor the role it may play in AD. We have generated *Drosophila* models to allow us to functionally characterize *Drosophila Ank2*, the ortholog of human *ANK1* and to determine its interaction with human Tau and Aβ. We show expression of human Tau 0N4R or the oligomerizing Aβ 42 amino acid peptide caused shortened lifespan, degeneration, disrupted movement, memory loss, and decreased excitability of memory neurons with co-expression tending to make the pathology worse. We find that *Drosophila* with reduced neuronal *Ank2* expression have shortened lifespan, reduced locomotion, reduced memory and reduced neuronal excitability similar to flies overexpressing either human Tau 0N4R or Aβ42. Therefore, we show that the mis-expression of *Ank2* can drive disease relevant processes and phenocopy some features of AD. Therefore, we propose targeting human ANK1 may have therapeutic potential. This represents the first study to characterize an AD-relevant gene nominated from EWAS.

## Introduction

Alzheimer’s disease (AD) is the most common form of dementia, with patients suffering from premature death and accelerated cognitive decline including memory loss. Post-mortem examination of AD brain samples reveals the accumulation of extracellular amyloid-beta (Aβ) plaques and intracellular neurofibrillary tangles (NFTs) of hyperphosphorylated microtubule associated protein Tau (MAPT), which is accompanied by gliosis, neuronal cell loss, and brain atrophy. Aβ is produced by the amyloidogenic cleavage of the amyloid precursor protein (APP) by β and γ secretases resulting in the formation of neurotoxic aggregating Aβ peptides with the 42 amino acid (aa) peptide (Aβ42) being more toxic than Aβ40 (Selkoe and Hardy, 2016). Tau exists in six different isoforms, which vary with the number of N-terminal domains (0N, 1N or 2N) and the number of C-terminal aggregating tubulin binding repeats (3R or 4R) (Arendt et al., 2016). The 4R isoforms are upregulated in AD brain and show stronger tubulin binding and aggregation than the 3R isoforms (Arendt et al., 2016). Another contributing factor to Tau aggregation is its hyperphosphorylation by a number of different kinases including glycogen synthase kinase-3β, cyclin-dependent kinase 5, JNK, microtubule-associated regulatory kinase, DYRK1a and CaMKII (Hanger et al., 1992; Ferrer et al., 2005; Plattner et al., 2006; Wang et al., 2007; Dolan and Johnson, 2010; Ghosh and Giese, 2015). It is not currently known how Aβ and Tau pathology are linked, however, the amyloid cascade hypothesis suggests that Aβ pathology leads to the other hallmarks of AD, including the spread of NFTs (Selkoe and Hardy, 2016) and recent work suggests changes in neuronal excitability and calcium (Ca^2+^) signaling may be important for their connection and disease progression (Spires-Jones and Hyman, 2014; Wu et al., 2016), but exactly how remains unknown.

Less than 5% of AD cases are due to autosomal dominant mutations in the *APP*, presenilin 1 (*PSEN1*) or *PSEN2* genes. The remainder of AD cases are sporadic, with incidence attributed to both genetic and environmental risk factors. Genome-wide association studies (GWAS) have identified a number of genes where common genetic variation is associated with increased risk of sporadic AD, including *APOE* (𝜀4 allele), *BIN1* and *PICALM* genes amongst others (Lambert et al., 2013) with many additional risk factors for AD being associated with lifestyle and/or the environment.

Epigenetics refers to the mitotically and meiotically heritable changes in gene expression without alterations in the underlying DNA sequence for instance by DNA methylation and downregulation of genes (Gräff and Sanchez-Mut, 2015). This also potentially allows for alterations in gene expression in response to environmental variation, such as stress, diet or exposure to environmental chemicals. In order to characterize the contribution of epigenetic mechanisms to AD etiology, recent epigenome-wide association studies (EWAS) of AD have been performed and have identified a number of genetic loci that are associated with increased risk of AD (De Jager et al., 2014; Lunnon et al., 2014; Smith et al., 2018). One locus that showed consistent cortical AD-associated hypermethylation in five independent cohorts resided in the *Ankyrin 1* (*ANK1*) gene (De Jager et al., 2014; Lunnon et al., 2014; Smith and Lunnon, 2017). A recent publication has demonstrated that *ANK1* DNA methylation in the entorhinal cortex is observed in only certain neurodegenerative diseases (Smith et al., 2019). This study reported disease-associated hypermethylation in AD, Huntington’s disease and to a lesser extent Parkinson’s disease (PD). The authors showed that disease-associated hypermethylation was only seen in donors with vascular dementia (VaD) and dementia with Lewy bodies (DLB) when individuals had co-existing AD pathology; in individuals with “pure” VaD or DLB, no *ANK1* hypermethylation was observed. ANK1 is an integral membrane and adaptor protein, that mediates the attachment of membrane proteins such as ion channels, cell adhesion proteins and receptors with the spectrin-actin cytoskeleton and is important for cell proliferation, mobility, activation, and maintenance of specialized membrane domains (Smith and Penzes, 2018).

Most of our understanding of the molecular changes that cause AD pathology comes largely from experiments using rodents to model genetic variation; however, these are models of familial AD, and do not recapitulate sporadic disease. As such, new drugs effective in these rodents have not translated to any new successful treatments for AD highlighting the need to generate and characterize new models of sporadic AD (McGowan et al., 2006; Crews and Masliah, 2010; Van Dam and De Deyn, 2011; Gama Sosa et al., 2012; Guo et al., 2012; De Jager et al., 2014). In *Drosophila*, neuronal overexpression of different human APP products (including Aβ42) and mutants has been reported to cause degeneration of the photoreceptor neurons of the fly eye, shortened lifespan, change in neuronal excitability as well as movement, circadian, sleep, and learning defects in a number of different studies (Iijima et al., 2004; Chiang et al., 2010; Speretta et al., 2012; Chen et al., 2014; Blake et al., 2015; Ping et al., 2015; Tabuchi et al., 2015). Likewise, neuronal overexpression of human Tau isoforms associated with AD have been shown to result in degeneration of the photoreceptor neurons of the fly eye, shortened lifespan, movement and learning defects in many different studies (Wittmann et al., 2001; Folwell et al., 2010; Iijima-Ando and Iijima, 2010; Kosmidis et al., 2010; Beharry et al., 2013; Papanikolopoulou and Skoulakis, 2015; Sealey et al., 2017). Fewer animal models have determined the effect of expression of human Aβ and Tau quantifying the effect on lifespan, axonal transport, synaptic morphology, degeneration of the eye and climbing (Folwell et al., 2010; Iijima et al., 2010; Lee et al., 2012; Shulman et al., 2014). Co-expression more accurately reflects the progression of AD in humans (Guerreiro and Hardy, 2011; Selkoe, 2012; Spires-Jones and Hyman, 2014; Bouleau and Tricoire, 2015; Arendt et al., 2016; Selkoe and Hardy, 2016). Furthermore, fewer studies have compared the effect of common variants nominated from GWAS for AD (Shulman et al., 2011, 2014; Gotz et al., 2012; Chapuis et al., 2013; Dourlen et al., 2016) and to our knowledge none from EWAS for AD. Thus, many genomic and epigenomic loci nominated in these studies remain uncharacterized in any living organism, with many of these genes or loci having a completely unknown function in the brain (Guerreiro and Hardy, 2011; Shulman et al., 2011; Gotz et al., 2012; De Jager et al., 2014; Del-Aguila et al., 2015; Devall et al., 2016).

In order to address these issues, we have characterized the first animal model to investigate the function of a locus nominated from EWAS in AD. We have investigated *ANK* mis-expression and compared its AD relevant phenotypes to *Drosophila* models expressing either (a) human mutant *APP* (which results in an aggregating form of oligomerized Aβ42), (b) MAPT (resulting in 0N4R Tau), (c) *APP* (Aβ42) with *MAPT* (0N4R Tau), and (d) *APP* (Aβ42) or *MAPT* (0N4R Tau) with *Ank* mis-expression, finding that mis-expression of these AD associated genes cause similar reduction in lifespan, movement, memory and neuronal excitability. We also report for the first time the effect of human Aβ42, 0N4R Tau and co-expression on 1 h memory and determine how Aβ42 and 0N4R Tau change mushroom body (MB) memory neuron excitability prior to neurodegeneration.

## Materials and Methods

### *Drosophila* Genetics

Flies were raised at a standard density with a 12 h:12 h light dark (LD) cycle with lights on at ZT 0 (Zeitgeber time) on standard *Drosophila* medium (0.7% agar, 1.0% soya flour, 8.0% polenta/maize, 1.8% yeast, 8.0% malt extract, 4.0% molasses, 0.8% propionic acid, and 2.3% nipagen) at 25°C. The following flies used in this study were previously described or obtained from the Bloomington and Vienna fly stock centers: wild type control was *Canton S w-* (*CSw-*) (gift from Dr. Scott Waddell, University of Oxford). Experimental genotypes were *elav-Gal4* (Bloomington stock center line number BL8760), *OK107-Gal4* (BL854), *GMR-Gal4* (BL9146), *uas-human MAPT (TAU 0N4R) wild type* (gift from Dr. Linda Partridge, University College London) (Wittmann et al., 2001; Kerr et al., 2011), *uas-human tandem Aβ42-22 amino acid linker-Aβ42* (gift from Dr. Damian Crowther, University of Cambridge) (Speretta et al., 2012), *uas-GFP* (gift from Dr. Mark Wu, Johns Hopkins University), *uas-GCaMP6f* [BL42747 (Shaw et al., 2018)], *uas-Ank2-RNAi* line A (BL29438), *uas-Ank2 RNAi* (Vienna *Drosophila RNAi* Center stock number VDRC107238 and KK10497) and *uas-Ank2* (gift from Dr. Ron R. Dubreuil, University of Illinois) (Mazock et al., 2010).

### Survival Assay

Approximately 2 days after eclosion 10 mated females were transferred to a vial containing standard food and maintained at 25°C throughout. Deaths were scored every 2 days and then transferred to a fresh food vial (Kerr et al., 2011). Data was presented as Kaplan–Meier survival curves with statistical analysis performed using log-rank tests to compare survival between genotypes. All statistical tests were performed using Prism (GraphPad Software Inc., La Jolla, CA, United States).

### Eye Degeneration Assay

Two to five day old adult flies were anesthetized by CO_2_ prior to immersion in ethanol in order to euthanize the fly to prevent any further movement during image capture (Folwell et al., 2010). The eyes were imaged with a Zeiss AxioCam MRm camera attached to a stereomicroscope (Zeiss SteREO Discovery.V8, up to 8× magnification). Surface area was quantified using Zeiss Zen software and normalized to the mean size of the wild type control. One-way ANOVA with Dunnett’s multiple comparisons was used to analyze data.

### Climbing Assay

Ten 2–5 day old flies were collected and given 1 h to acclimatize to the test vial in an environmentally controlled room at 25°C and 70% humidity. Using the negative geotaxis reflex of *Drosophila*, flies were gently tapped to the bottom of a 7.5 cm plastic vial and the number of flies that crossed a line drawn 2 cm from the top of the tube in 10 s was counted, and then expressed as a % which was referred to as the climbing performance or climbing index (Iijima et al., 2004; Sun et al., 2018). One-way ANOVA with Dunnett’s multiple comparisons was used to analyze data.

### Memory

One hour memory and sensory controls were performed as previously described using the olfactory-shock aversive conditioning assay (Cavaliere et al., 2013; Malik et al., 2013; Malik and Hodge, 2014). Experiments were performed with groups of 30–50 flies aged between 2 and 5 days old of a given genotype, in a T-maze apparatus housed in an environmentally controlled room at 25°C and 70% humidity under dim red light. Flies were exposed to either 4-methylcyclohexanol (MCH, Sigma, ∼1:200) or 3-octanol (OCT, Sigma, ∼1:100) diluted in mineral oil (Sigma) paired with 1.5 s pulses of 60 V electric shock interspersed with 3.5 s pauses from shock for a minute. After 30 s of fresh air the flies were exposed to the reciprocal order without shock for another minute. After a 1 h rest, memory was assessed by transferring the flies to a choice point of the T maze, with one arm containing the shock paired odor and the other the non-shock paired odor, flies showed learning by avoiding the shock paired odor (i.e., correct flies).

$$\begin{array}{c} \mathrm{Performance}\;\mathrm{index}\;\left(\mathrm{PI}\right)\;=\;(\mathrm{number}\;\mathrm{of}\;\mathrm{correct}\;\mathrm{flies}\;-\;\mathrm{number}\;\mathrm{of}\;\mathrm{incorrect}\;\mathrm{flies})/\mathrm{total}\;\mathrm{number}\;\mathrm{of}\;\mathrm{flies} \end{array}$$

To eliminate odor bias, the assay was performed with two groups of flies (30–50 flies in each), one shocked with MCH and then the other shocked with OCT. The average of the two groups was taken to give an *n* = 1 PI value. Control experiments were performed to show that the different genotypes of flies could respond to MCH, OCT and shock alone. One-way ANOVA with Dunnett’s multiple comparisons was used to analyze data.

### Calcium Imaging and Microscopy

Calcium imaging and imaging of the MB structure was performed using a genetically encoded GCaMP Ca^2+^ reporter and adapting previously published protocols (Cavaliere et al., 2012; Gillespie and Hodge, 2013; Malik et al., 2013; Schlichting et al., 2016; Shaw et al., 2018). Adult flies of the indicated genotypes using *OK107-Gal4* and *uas-GCaMP6f* were collected between 2 and 5 days post-eclosion, decapitated and the brain dissected in extracellular saline solution containing (in mM): 101 NaCl, 1 CaCl_2_, 4 MgCl_2_, 3 KCl, 5 glucose, 1.25 NaH_2_PO_4_, 20.7 NaHCO_3_, pH adjusted to 7.2. Brains were placed ventral side up in the recording chamber, secured with a custom-made anchor and continuously perfused with aerated saline. Images of the whole MB of each genotype was captured in the resting state. To activate neurons, high concentration KCl (100 mM in saline) was bath applied through the perfusion system for 4 min and then washed out. The calcium fluorescence signal was acquired using a CCD camera (Zeiss Axiocam) and a 470 nm LED light source (Colibri, Zeiss) on an upright Zeiss Examiner microscope with a 20× water immersion lens, recorded with ZEN (Zeiss, 4 frames/sec) and plotted with Microsoft Excel. One-way ANOVA with Dunnett’s multiple comparisons was used to analyze data.

## Results

### Mis-Expression of Human Mutant APP, MAPT or Ank2 Shortens Lifespan

**FIGURE 1:**
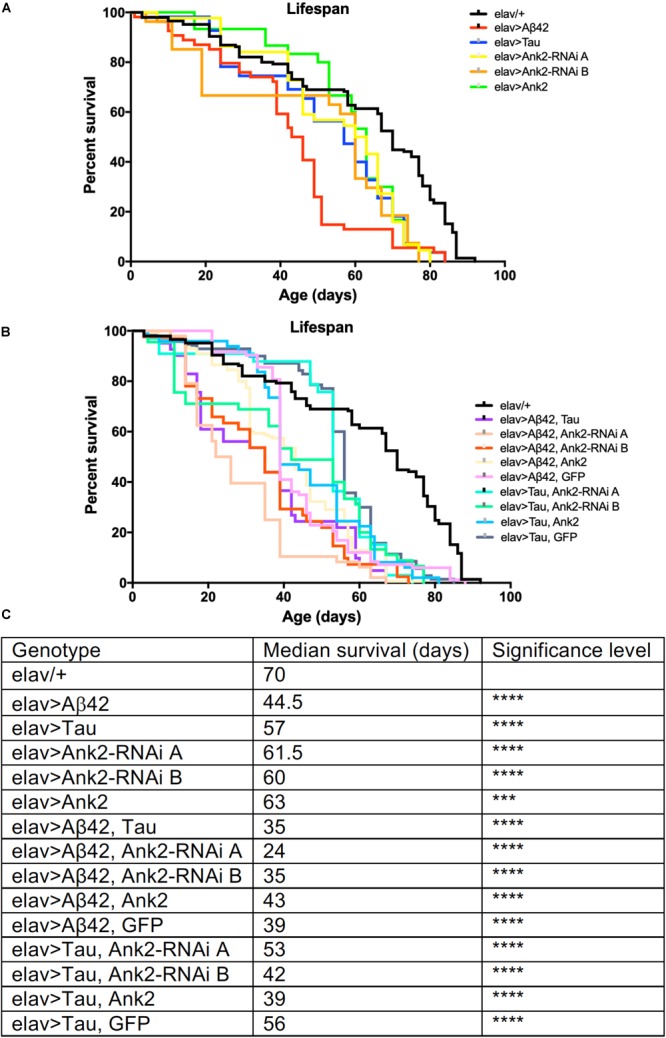
The effect of expression of human mutant *APP* (Aβ42), *MAPT* (Tau 0N4R) and *Drosophila Ank2* on *Drosophila* lifespan. **(A)** Survival curves of pan-neuronal expressing Aβ42 (*elav > Aβ42*), Tau (*elav > Tau*), *Ank2-RNAi* (*elav > Ank2-RNAi line A or B*), Ank2 (*elav > Ank2*), **(B)** Tau with Aβ42 (*elav > Tau, Aβ42*), Aβ42 with *Ank2-RNAi* (*elav > Aβ42, Ank2-RNAi line A or B*), Aβ42 with Ank2 (*elav > Aβ42, Ank2*), Aβ42 with Ank2 (*elav > Aβ42, Ank2*), Aβ42 with Ank2 (*elav > Aβ42, GFP*), Tau with *Ank2-RNAi* (*elav > Tau, Ank2-RNAi line A or B*), Tau with Ank2 (*elav > Tau, Ank2*), Tau with GFP (*elav > Aβ42, GFP*) compared to wild type control (*elav/+*) flies kept at 25°C. Misexpression of all Alzheimer’s disease (AD) genes caused a significant reduction in lifespan compared to control using the Kaplan–Meier and log rank test (*n* > 100 per genotype of flies). **(C)** Table listing all genotypes characterized with median lifespan (days) and significant reductions in lifespan as determined by Kaplan–Meier and log rank test and indicated as ^∗^*p* < 0.05, ^∗∗^*p* < 0.01, ^∗∗∗^*p* < 0.001, ^∗∗∗∗^*p* < 0.0001 and used in all subsequent figures.

Increased neuronal levels of Aβ or Tau lead to AD pathology and early death (Selkoe, 2012; Selkoe and Hardy, 2016). We therefore overexpressed in all neurons either an aggregating form of human mutant *APP* that encodes oligomerized human Aβ42 [tandem Aβ42 (Speretta et al., 2012)] or human MAPT [0N4R Tau (Papanikolopoulou and Skoulakis, 2015; Sealey et al., 2017)] both of which caused premature death reducing the flies’ lifespan by about 25% (Figure 1A,C). In order to test for a genetic interaction between the two neurotoxic genes, we overexpressed both mutant *APP* (Aβ42) and *MAPT* (0N4R) together in all neurons and got a further reduction to exactly half the lifespan of a normal fly. The reduction in median lifespan due to co-expression was equivalent to an additive effect of the shortening of life due to mutant *APP* (Aβ42) and *MAPT* (0N4R) alone, this suggests that Aβ42 and Tau pathology may act in separate pathways to cause neurotoxicity and early death.

**FIGURE 2:**
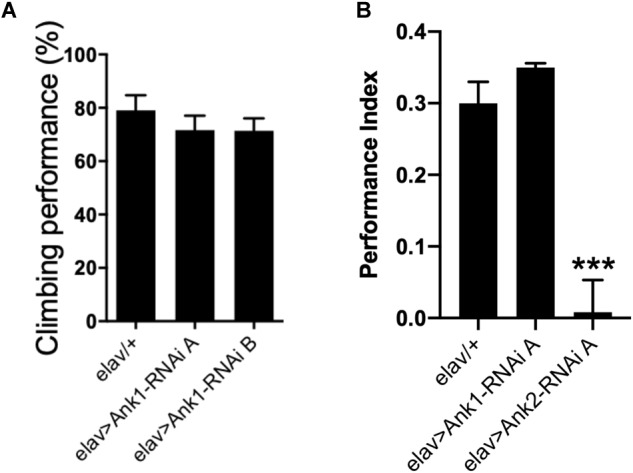
The effect of pan-neuronal expression of *Drosophila Ank1* and *Ank2* on behavior. **(A)** The negative geotaxis-climbing reflex was used to quantify motor deficits of 2–5 day old flies. Pan-neuronal reduction of *Ank1* (*elav-Gal4 > Ank1-RNAi line A or B*) was compared to control using one-way ANOVA with Dunnett’s multiple comparison and showed no significant difference. *n* ≥ 6 groups of 10 flies. **(B)** One hour memory of 2–5 day old flies was assessed using the olfactory shock-conditioning assay. Pan-neuronal reduction in Ank2 (*elav > Ank2-RNAi line A*) as opposed to Ank1 (*elav > Ank1-RNAi line A*) caused a significant reduction in memory compared to control using one-way ANOVA with Dunnett’s multiple comparisons. Error bars are standard error of the mean (SEM). *n* ≥ 4 groups of ∼50 flies.

Recently, human *ANK1* gene has been shown to be differentially methylated in AD (Lunnon et al., 2014). There are two other *ANK* genes in the human genome, *ANK2*, and *ANK3*. In *Drosophila*, there are two *Ank* genes, the ubiquitously expressed *Ank1*, and neuron specific *Ank2* (Koch et al., 2008; Pielage et al., 2008; Mazock et al., 2010; Keller et al., 2011; Siegenthaler et al., 2015). The closest ortholog to *Drosophila Ank1* is human *ANK3* with 51% total amino acid identity while the closest ortholog of *Drosophila Ank2* is human *ANK1* with 43% amino acid identity. Human *ANK2* is more similar to *Drosophila Ank1* (49% identity) than *Drosophila Ank2* (33% identity). Because hypermethylation of human *ANK1* has been reported in AD cortex (Lunnon et al., 2014), we used *RNAi* to knock-down expression of *Ank1* and *Ank2* comparing the effect of reducing these genes in all *Drosophila* neurons using two different *RNAi* transgenes designed to non-overlapping regions of each gene. Pan-neural reduction in expression of *Drosophila Ank1* did not cause any AD relevant behavioral deficits (Figure 2A,B), while reduction in *Ank2* using the same promoter and assay did cause a reduction in 1 h memory (Figure 2B). Therefore, we conclude, that *Drosophila Ank2* is the closest functional ortholog of human *ANK1*, which we characterized in subsequent experiments.

We found that pan-neural reduction of *Ank2* resulted in a shortening of lifespan by 15% compared to control (Figure 1B,C). We verified our results by using two independent *RNAi* lines (A and B) to non-overlapping sequences in *Ank2*, both lines gave similar results in all assays. Depending on the genomic location of DNA methylation, it can result in either increased or decreased expression of the target gene (Suzuki and Bird, 2008); we therefore tested if neuronal *Ank2* overexpression affected longevity and found that it also shortened lifespan (∼10%), but at a lower significance level to all other genotypes. In order to test if there was a genetic interaction between mutant *APP* (Aβ42) or *MAPT* (0N4R) and *Ank2* we generated flies that co-expressed mutant *APP* (Aβ42) or *MAPT* (0N4R) with the *Ank2* transgenes. We found that the detrimental effect of each gene on lifespan remained (Figure 1B,C), suggesting changing the level of fly Ank2 could not rescue the shortened lifespan caused by overexpression of human mutant *APP* (Aβ42) or *MAPT* (0N4R) to a level similar to wild type control (*elav/+*). One potential cause of a reduction or suppression of toxicity of a gene product is dilution of Gal4. This is due to a single Gal4 transcription factor driving expression by binding to a single UAS transgene [e.g., mutant *APP* (Aβ42) or *MAPT* (0N4R)] which may get diluted when adding a second UAS site of a gene (e.g., *Ank2*). This results in a reduction in the amount of the AD toxic gene product being made, giving a false positive of a suppression phenotype. To control for such a dilution effect, we generated flies that co-expressed a second unrelated neutral gene product (GFP) with either human mutant *APP* (Aβ42) or *MAPT* (0N4R Tau). There was no significant change in neurotoxicity of the mutant *APP* (Aβ42) or *MAPT* (0N4R Tau) when expressed alone or with GFP.

### Overexpression of Human Mutant APP or Mutant MAPT but Not Mis-Expression of Ank2 Caused Degeneration of Photoreceptor Neurons

**FIGURE 3:**
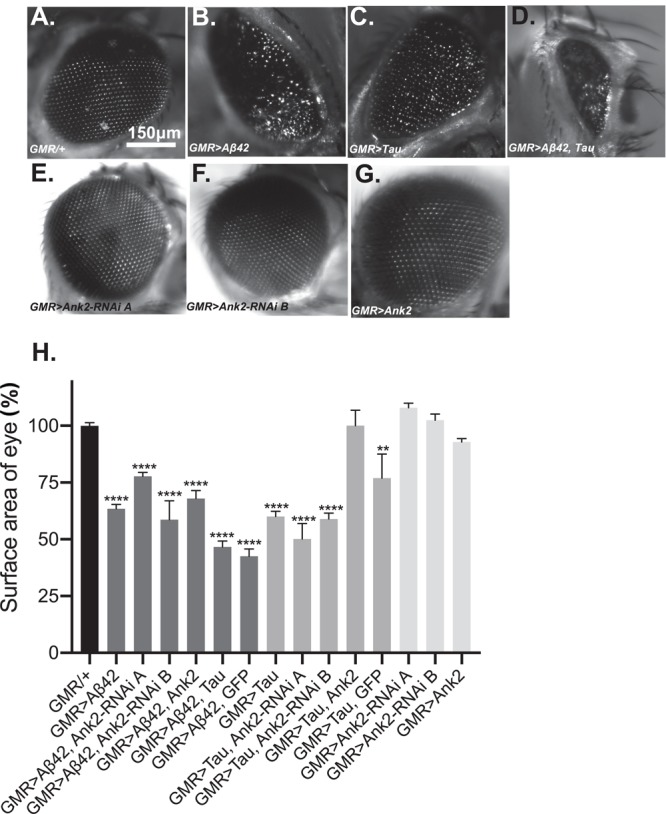
The effect of expression of human mutant *APP* (Aβ42), *MAPT* (Tau 0N4R), and *Drosophila Ank2* on degeneration of photoreceptor neurons. Images of 2–5 day old compound eyes of **(A)** control fly (*GMR-Gal4/+*) showing the regular alignment of ommatidia compared to photoreceptors neurons overexpressing, **(B)** Aβ42 (*GMR-Gal4 > Aβ42*), **(C)** Tau (*GMR-Gal4 > Tau*), and **(D)** Tau with Aβ42 (*GMR-Gal4 > Tau, Aβ42*) which were smaller and displayed a “rough eye” phenotype. **(E,F)** photoreceptors expressing *Ank2-RNAi* (*GMR > Ank2-RNAi line A or B*) or **(G)** Ank2 (*GMR > Ank2*) appeared normal. **(H)** Degeneration of photoreceptor neurons was quantified as normalized percentage surface area of the eye of genotypes compared to the mean of the control (*GMR/+*) which was set at 100%, comparisons were made between to mutant genotypes compared to control using one-way ANOVA with Dunnett’s multiple comparisons. Genotypes that included expression of *APP* (Aβ42) or *MAPT* (Tau 0N4R) in the eye showed a significantly reduced size of eye, while those mis-expressing *Drosophila Ank2* did not. Co-expression of *MAPT* (Tau 0N4R) and *Ank2* rescued eye size to a level indistinguishable from wild type. Error bars are SEM. *n* ≥ 7 eyes per genotype.

Increased levels of aggregating toxic Aβ and Tau lead to neurodegeneration and AD. In order to model this neurotoxicity in *Drosophila*, human mutant *APP* causing Aβ42 production (Figure 3B) or mutant MAPT (0N4R Tau) (Figure 3C) was expressed throughout development and adulthood in the photoreceptor neurons of the eye, resulting in a so called “rough eye” phenotype, where the degeneration and loss of the normally regularly arrayed ommatidia of the compound eye of wild type flies (Figure 3A) gives rise to a disorganized and smaller eye. The loss of photoreceptors could be quantified by measuring the total surface area of the eye, which showed that expression of human mutant *APP* (Aβ42) and *MAPT* (0N4R Tau) reduced the size of the eye by 37% and 40%, respectively (Figure 3H). Co-expression of human mutant *APP* (Aβ42) and *MAPT* (0N4R Tau) (Figure 3D,H), resulted in a 53% reduction in eye size, suggesting that Aβ42 and Tau act in partially overlapping pathways to cause neurotoxicity in the fly eye. Reduction of *Ank2* (Figure 3E,F), overexpression of *Ank2* (Figure 3G) and co-expression of *Ank2* and *Tau* did not cause degeneration or reduction in size of the eye (Figure 3H), although the latter cannot be considered a rescue as although the eye was significantly larger (*p* < 0.01) than eyes expressing Tau it was not significantly bigger than those expressing Tau and GFP suggesting any suppression maybe due to a Gal4 dilution effect.

### Overexpression of Human Mutant APP and MAPT Genes and Reduction in Ank2 Expression Caused Locomotor Deficits

**FIGURE 4:**
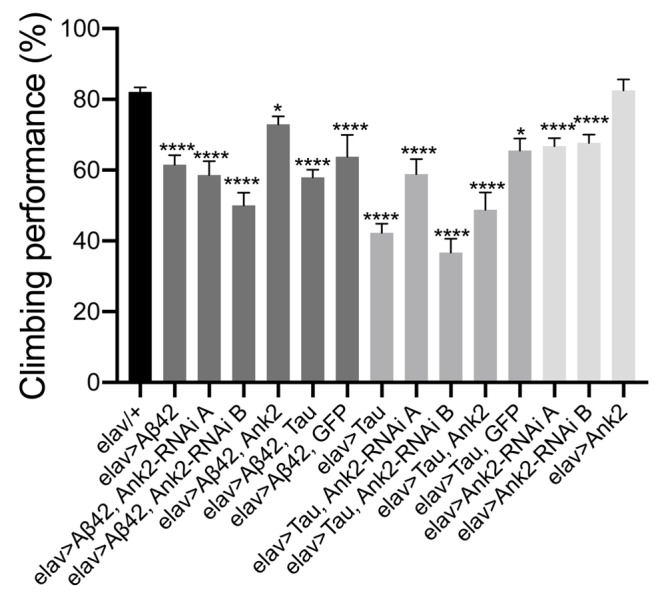
The effect of expression of human mutant *APP* (Aβ42), *MAPT* (Tau 0N4R) or *Drosophila Ank2* on locomotor behavior. The negative geotaxis-climbing reflex was used to quantify locomotor behavior of 2–5 day old flies. About 80% of control (*elav-Gal4/+*) flies were able to climb to the top of test vial within 10 s; this compared to experimental genotypes expressing AD transgenes throughout their nervous system. Mis-expression of all AD associated genes except for overexpression of *Ank2* alone, resulted in a significant reduction in climbing performance using one-way ANOVA with Dunnett’s multiple comparisons. Error bars are SEM. *n* ≥ 15 groups of 10 flies.

Flies exhibit a negative geotaxis reflex, such that after tapping on a surface ∼80% of 2–5 day old wild type flies will climb to the top of a tube in 10 s. This startle-induced reflex requires the co-ordinated activity of dopaminergic neurons afferent to the MB, and of MB Kenyon cells, in addition to MB efferent neurons which communicate with central motor centers (Sun et al., 2018). In order to quantify any detrimental effect of *human mutant APP* (Aβ42) and *MAPT* (0N4R Tau) on this behavior, we pan-neuronally (*elav-Gal4* expressed *human mutant APP* (Aβ42) and *MAPT* (0N4R Tau), which resulted in a 20% and 40% reduction in climbing ability, respectively. Likewise, reduction in Ank2 expression caused a 15% reduction in climbing, while Ank2 overexpression had no effect. All other gene combinations were found to significantly reduce gross climbing performance (Figure 4).

### Overexpression of Human Mutant APP and MAPT Genes and Reduced Ank2 Causes Memory Deficits

**FIGURE 5:**
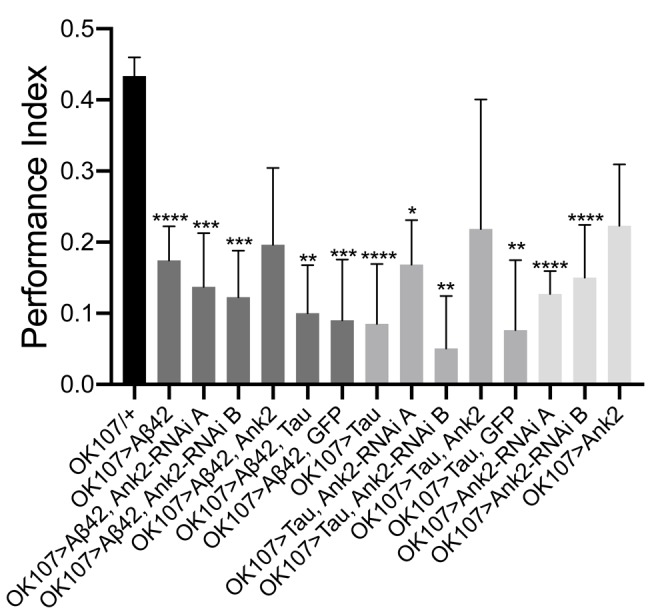
The effect of expression of human mutant *APP* (Aβ42), *MAPT* (Tau 0N4R) and *Drosophila Ank2* on memory. One hour memory of 2–5 day old flies was assessed using the olfactory shock-conditioning assay. The performance index of flies expressing AD transgenes throughout their mushroom body (MB) was compared to control (*OK107-Gal4/+*) and were found to show a significant reduction in memory using one-way ANOVA with Dunnett’s multiple comparisons, except genotypes overexpressing *Ank2*. Error bars are SEM. *n* ≥ 4 groups of ∼100 flies.

As multiple forms and phases of memory, including anterograde memory, are affected by AD (Walsh and Selkoe, 2004), therefore we measured the effect of the human mutant *APP* (Aβ42), *MAPT* (0N4R Tau) and *Drosophila Ank2* on memory. We performed the olfactory shock assay on 2–5 day old flies (Cavaliere et al., 2013; Malik et al., 2013; Malik and Hodge, 2014) assessing associative memory at the 1 h time point, which is considered to be intermediate memory in *Drosophila*. Olfactory shock memory is mediated by MB neurons and we therefore drove expression of the genes using a promoter (*OK107-Gal4*) with broad expression in these neurons (Cavaliere et al., 2013; Malik et al., 2013). We found MB expression of human mutant *APP* (Aβ42) or *MAPT* (0N4R Tau) caused a large reduction in memory (Figure 5). *Ank2* is highly expressed in the adult MB as shown by *Ank2* reporter line (*R54H11-Gal4*) expression (Pfeiffer et al., 2008) and previous studies (Siegenthaler et al., 2015). Reduction, as opposed to overexpression, of *Ank2* also caused a reduction in memory (Figure 5). Co-expression of human mutant *APP* (Aβ42) and *MAPT* (0N4R Tau; Figure 5) or either AD gene with *Ank2-RNAi* caused a similar reduction in memory, suggesting that all three genes may act in the same pathway in MB neurons. Overexpression of *Ank2*, or co-expression of *Ank2* with either human mutant *APP* (Aβ42) or *MAPT* (0N4R Tau) did not cause a significant reduction in memory. The latter cannot be considered rescue of the memory deficit caused by expression of Aβ42 or Tau alone, as the memory of *Ank2* co-expressed with either human mutant *APP* (Aβ42) or *MAPT* (0N4R Tau) was not significantly greater than that of human mutant *APP* (Aβ42) or *MAPT* (0N4R Tau) co-expressed with GFP, again suggesting a Gal4 dilution effect was contributing to the rescue of the mutant phenotype.

**FIGURE 6:**
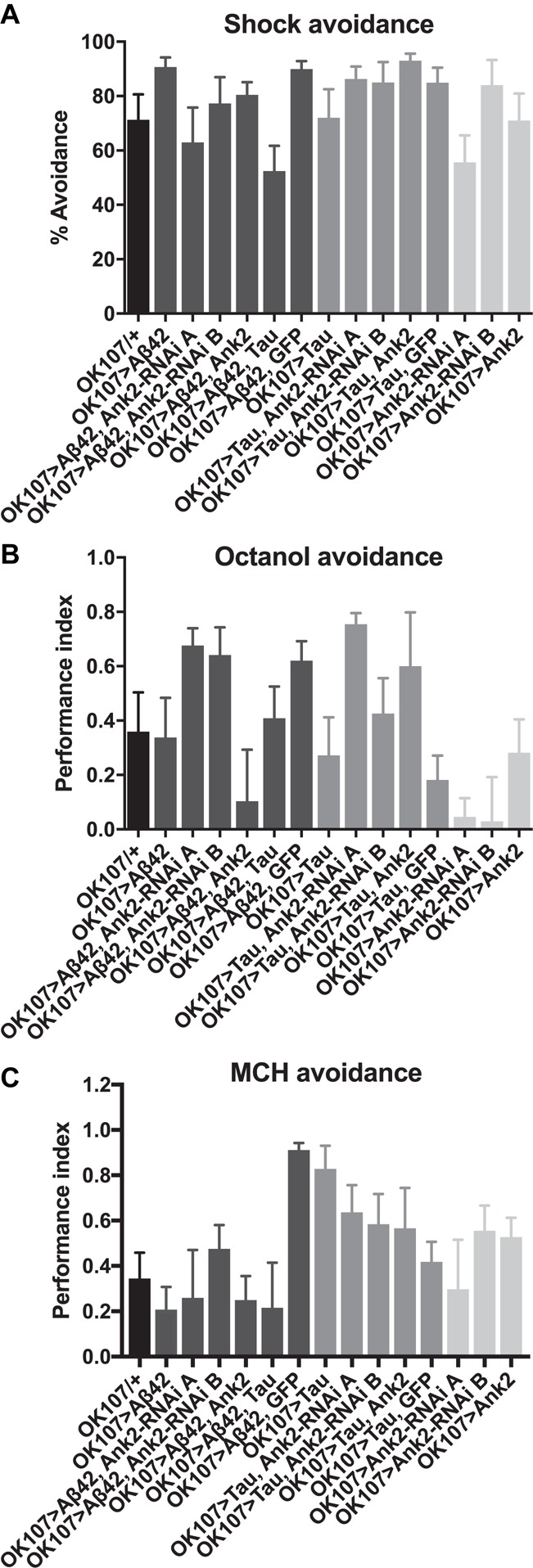
The effect of expression of human mutant *APP* (Aβ42), *MAPT* (Tau 0N4R) and *Drosophila Ank2* on response to negative reinforcement (shock) and olfaction. **(A)** The response of 2–5 day old flies to negative reinforcement was quantified as % avoidance of shock, with experimental genotypes with AD genes expressed throughout their MB compared to control (*OK107/+*). The avoidance of these genotypes of flies to 3-octanol **(B)** and methylcyclohexanol (MCH) **(C)** odors was expressed as a performance index. All genotypes responded to odors and shock in a manner not significantly different from wild type using one-way ANOVA with Dunnett’s multiple comparisons. *n* ≥ 4 groups of ∼50 flies.

For flies to be able to perform the olfactory shock assay, the fly must be able to respond normally to shock and the odors used in the memory task. Therefore, we performed behavioral controls on 2–5 day old flies that showed that there was no significant difference between mutant genotypes and wild type in terms of avoidance of electric shock (Figure 6A), octanol (Figure 6B) and methylcyclohexanol (MCH; Figure 6C) odors (Cavaliere et al., 2013; Malik et al., 2013; Malik and Hodge, 2014). Therefore, any mutant genotype showing a significant reduction in memory in Figure 5 was a bona fide memory mutant.

### Mis-Expression of Human Mutant APP (Aβ42), MAPT (0N4R Tau), and Ank2 Decreased the Neural Excitability of Memory Neurons Prior to Any Observable Neurodegeneration

**FIGURE 7:**
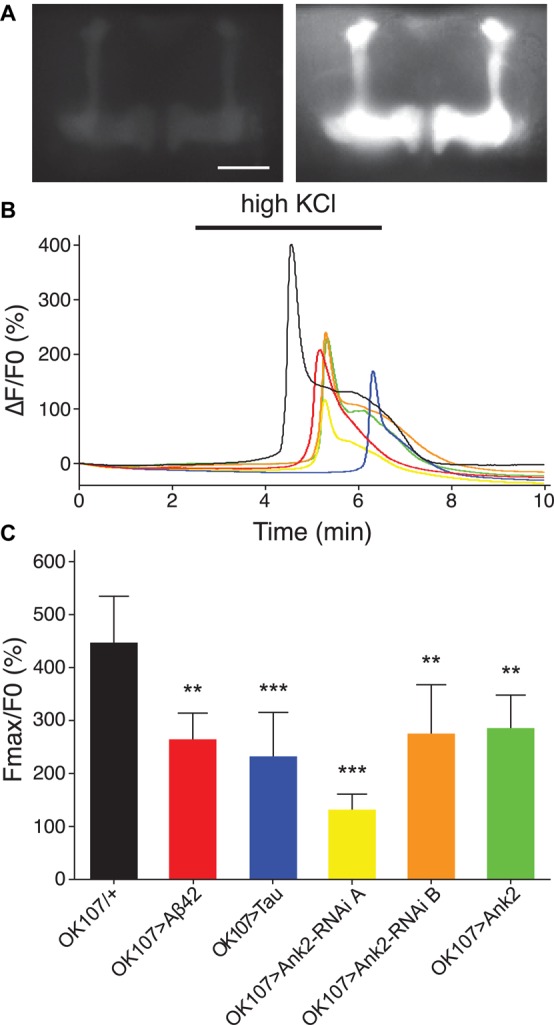
The effect of expression of human mutant *APP* (Aβ42), *MAPT* (Tau 0N4R), and *Drosophila Ank2* on activation of mushroom body neurons. **(A)** Exemplary control 2–5 day old fly MB expressing the calcium reporter GCaMP6f shows a big increase in relative fluorescence in response to elevated KCl (100 mM, bath-applied). Images show the same brain before (left) and during (right) KCl application; scale bar 50 μm. **(B)** The increase in neuronal activity is expressed as the relative change in fluorescence (ΔF/F0) over time showing that high KCl (indicated by bar) causes peak Ca^2+^ influx that returns to baseline after washing. **(C)** Quantitative analysis of the maximal response for the indicated genotypes shows a reduced responsiveness of all mutants. Data was analyzed with one-way ANOVA with Tukey’s *post hoc* and error bars are standard deviation, *n* ≥ 5 brains.

**FIGURE 8:**
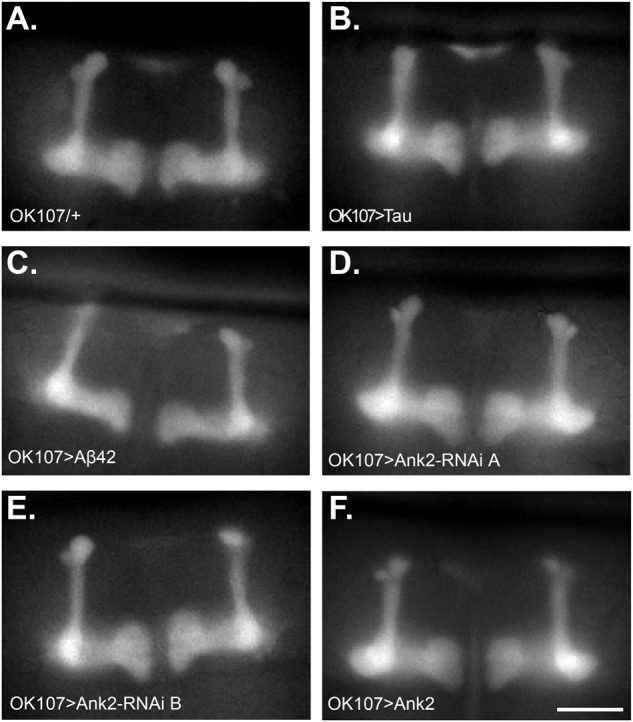
Mushroom body morphology is not affected by expression of human mutant *APP* (Aβ42), *MAPT* (Tau 0N4R), and *Drosophila Ank2* in young flies. Images show representative mushroom bodies of 2–5 day old flies visualized by expression of the Ca^2+^ reporter GCaMP6f of **(A)** a control fly (*OK107/+*), **(B)** *OK107 > Tau*, **(C)** *OK107 > Aβ42*, **(D)** *OK107 > Ank2-RNAi line A*, **(E)** *OK107 > Ank2-RNAi line B*, and **(F)** *OK107 > Ank2* which all show the regular organization of α, β, γ, α′ and β′ lobes. Scale bar is 50 μm.

Changes in neuronal excitability and Ca^2+^ signaling are thought to occur early in disease progression prior to neurodegeneration and are proposed to mediate early changes in behavior in AD, such as memory loss (Spires-Jones and Hyman, 2014; Wu et al., 2016). Therefore, in order to determine how mis-expression of human mutant *APP* (Aβ42), *MAPT* (0N4R Tau), and *Ank2* may lead to changes in neuronal function and AD relevant phenotypes, we expressed the genetically encoded Ca^2+^ reporter, GCaMP6f using the same *OK107-*Gal4 MB promoter as for the memory experiments and then measuring peak intracellular Ca^2+^ in response to high [K^+^] solution that non-specifically depolarizes neurons (Malik et al., 2013). The axons and synaptic terminals of MB neurons form a pair of bilaterally arranged and symmetrically lobed structures which show low basal Ca^2+^ fluorescence levels (Figure 7A left panel) which, in response to depolarizing high [K^+^] saline, show a large peak in Ca^2+^ fluorescence levels (Figure 7A right panel). The increase in neuronal activity can be expressed as the relative fluorescence change (ΔF/F0) over time (Figure 7B) showing that high K^+^ causes a rapid peak Ca^2+^ influx that returns to baseline after washing. MB wide overexpression of human mutant *APP* (Aβ42), *MAPT* (0N4R Tau) or mis-expression of *Ank2* all caused a similar decrease in peak Ca^2+^ influx of memory neurons (Figure 7C), suggesting a reduction in neuronal excitability. A previous study showed human 0N4R tau expression in γ MB neurons reduced learning and 1.5 h memory in 3–5 day old young flies which were demonstrated to still have their γ neurons intact. However, by day 45, the 0N4R expressing γ neurons had started to neurodegenerate (Mershin et al., 2004). In order to verify the MB were intact in our 2–5 day old flies overexpressing human mutant *APP* (Aβ42), *MAPT* (0N4R Tau) or mis-expressing fly *Ank2* throughout the MB we imaged the MB with GCaMP6f and saw that all the genotypes had intact MB α, β, γ, α′, and β′ lobes (Figure 8).

## Discussion

In this study, we have characterized the first animal model based on mis-expression of the AD EWAS nominated *ANK1* gene ortholog in *Drosophila*. Altered DNA methylation of human *ANK1* occurs in AD brains, especially in regions that show gross AD pathology, such as the entorhinal cortex (De Jager et al., 2014; Lunnon et al., 2014). Here, we found reduction in expression of the fly ortholog of human *ANK1*, which is *Drosophila Ank2*, in areas of the brain responsible for memory (the MB), caused memory impairment similar in magnitude to that caused by overexpression of human mutant *APP* resulting in an oligomerizing form of Aβ42 or *MAPT* producing the 0N4R isoform of Tau, both of which are particularly associated with the disease. In flies, overexpression of fly *Ank2*, did not result in degeneration of the eye, locomotor or memory defects but did lead to mild shortening of lifespan (by ∼10%) and reduced MB excitability.

In addition to memory loss, animals with reduced neuronal *Ank2* also recapitulated the shortening of lifespan seen in those with AD, again a phenotype seen in flies overexpressing human mutant *APP* (Aβ42) or *MAPT* (0N4R Tau). Co-expression of human mutant *APP* (Aβ42) and *MAPT* (0N4R Tau) caused a further reduction in lifespan, suggesting that the two molecules may act in partially non-overlapping and therefore additive pathways that lead to the pathology causing the flies to die early. A similar effect on lifespan has been reported previously for another form of *APP* that resulted in a non-oligomerizing version of Aβ42 and another form of *MAPT*, resulting in the 1N4R isoform of Tau, however, the longevity assays were only run for roughly half the lifespan of the flies (Folwell et al., 2010). We also found a reduction in locomotion in flies expressing human mutant *APP* (Aβ42), *MAPT* (0N4R Tau) and reduced *Ank2*. Interestingly, a recent study has shown that *ANK1* hypermethylation is observed in PD, a movement disorder, that can also be associated with dementia (Smith et al., 2019). In the eye, overexpression of human mutant *APP* (Aβ42) or *MAPT* (0N4R Tau) were both neurotoxic causing degeneration of the fly eye photoreceptor neurons. Co-expression caused a further reduction implying the two act in separate and additive pathways to cause neurotoxicity and neuronal death. Reduction in *Ank2* did not cause degeneration of the eye suggesting that *Ank2* may affect neuronal function independent of degeneration. This is likely to be via changes in neuronal excitability, as we saw when the gene was misexpressed in MB neurons (Smith and Penzes, 2018). We found misexpression of human mutant *APP* (Aβ42), *MAPT* (0N4R Tau) and *Ank2* all reduced the peak Ca^2+^ response of MB neurons, a decrease in excitability likely to contribute to the memory deficits of these flies.

In *Drosophila, Ank2* has been shown to be important for synaptic plasticity and stability (Koch et al., 2008; Pielage et al., 2008; Massaro et al., 2009; Keller et al., 2011; Bulat et al., 2014) and is involved in a glia mediated pathway that causes degeneration of motor neurons (Keller et al., 2011). Therefore, it is possible that reduction of *Ank2* may only cause degeneration in certain types of neurons. Furthermore, because human *ANK1* has also been shown to be misexpressed in glia in the AD brain (Mastroeni et al., 2017), it is also possible that neurodegeneration results from misexpression of *Ank2* in glia.

We do not know how Ankyrin interacts with Tau and what changes in Tau protein level, location or phosphorylation result from *Ankyrin* misexpression. We know that Ankyrins are adaptor proteins that attach to integral membrane proteins such as the Na^+^/K^+^ ATPase, voltage-gated Na^+^ (Na_v_1), voltage-gated K^+^ (KCNQ) and TrpA1 channels, and then link them to the actin-spectrin based membrane cytoskeleton, with some of these molecules having been associated with AD (Bennett and Baines, 2001; Yamamoto et al., 2007; Smith and Penzes, 2018). Therefore the loss of Ankyrin might be predicted to disrupt the proper clustering of these ion channels, some of which are involved in MB-memory and calcium signaling (Cavaliere et al., 2013), and hence may result in the decreased neuronal excitability detected by our MB calcium imaging experiments and which might be required for proper memory formation. The changes in calcium influx may also cause dysregulation of calcium dependent phosphorylation of Tau, also known to disrupt MB dependent memory and shorten lifespan (Papanikolopoulou and Skoulakis, 2015). Finally, the loss of Ankyrin may distrupt the actin-spectrin cytoskeleton, which may result in pathological changes in microtubules exacerbated by their association with Tau. More experiments are required to investigate the nature of the interaction between Ankyrin and Tau in AD.

In summary, we have characterized the first animal model of a gene implicated in AD that was nominated from EWAS. We found that mis-expression of the fly ortholog of this gene, *Ank2*, in central neurons caused a range of AD-relevant phenotypes such as shortened lifespan, memory loss, and changes in neuronal excitability similar to those resulting from human Aβ42 and 0N4R Tau. In alignment with previous AD studies, we conclude that *Ank2* – or, in humans, *ANK1* – likely plays a role in AD neuropathology.

## Author Contributions

JH, BM, EB, JD, AO, and JJLH performed the experiments. JH devised the experiments and wrote the manuscript. JJLH and KL secured the funding. JH, BM, EB, and KL edited the drafts of the manuscript.

## Conflict of Interest Statement

The authors declare that the research was conducted in the absence of any commercial or financial relationships that could be construed as a potential conflict of interest.
